# Covariance of Sun and Shade Leaf Traits Along a Tropical Forest Elevation Gradient

**DOI:** 10.3389/fpls.2019.01810

**Published:** 2020-01-31

**Authors:** Roberta E. Martin, Gregory P. Asner, Lisa Patrick Bentley, Alexander Shenkin, Norma Salinas, Katherine Quispe Huaypar, Milenka Montoya Pillco, Flor Delis Ccori Álvarez, Brian J. Enquist, Sandra Diaz, Yadvinder Malhi

**Affiliations:** ^1^School of Geographical Sciences and Urban Planning, Arizona State University, Tempe, AZ, United States; ^2^Center for Global Discovery and Conservation Science, Arizona State University, Tempe, AZ, United States; ^3^Department of Biology, Sonoma State University, Rohnert Park, CA, United States; ^4^Environmental Change Institute, School of Geography and the Environment, University of Oxford, Oxford, United Kingdom; ^5^Sección Química, Pontificia Universidad Católica del Perú, Lima, Perú; ^6^Departamento Académico de Biología, Universidad Nacional de San Antonio Abad del Cusco, Cusco, Perú; ^7^Department of Ecology and Evolutionary Biology, University of Arizona, Tucson, AZ, United States; ^8^The Santa Fe Institute, Santa Fe, NM, United States; ^9^Instituto Interdisciplinario de Biología Vegetal (CONICET-UNC) y FCEFyN, Universidad Nacional de Córdoba, Córdoba, Argentina

**Keywords:** canopy chemistry, sun-shade adjustment, plant functional traits, community assembly, Andes-Amazon, Peru, Spectranomics

## Abstract

Foliar trait adaptation to sun and shade has been extensively studied in the context of photosynthetic performance of plants, focusing on nitrogen allocation, light capture and use *via* chlorophyll pigments and leaf morphology; however, less is known about the potential sun-shade dichotomy of other functionally important foliar traits. In this study, we measured 19 traits in paired sun and shade leaves along a 3,500-m elevation gradient in southern Peru to test whether the traits differ with canopy position, and to assess if relative differences vary with species composition and/or environmental filters. We found significant sun-shade differences in leaf mass per area (LMA), photosynthetic pigments (Chl ab and Car), and δ^13^C. Sun-shade offsets among these traits remained constant with elevation, soil substrates, and species compositional changes. However, other foliar traits related to structure and chemical defense, and those defining general metabolic processes, did not differ with canopy position. Our results suggest that whole-canopy function is captured in many traits of sun leaves; however, photosynthesis-related traits must be scaled based on canopy light extinction. These findings show that top-of-canopy measurements of foliar chemistry from spectral remote sensing approaches map directly to whole-canopy foliar traits including shaded leaves that cannot be directly observed from above.

## Introduction

Solar radiation is one of the most limiting resources in tropical forests (Denslow, 1987; Chazdon et al., 1996; King et al., 2005; Smith et al., 2019). Evolution by natural selection has resulted in a diverse set of strategies within and across tropical tree species to maximize light interception and utilization. One strategy to achieve this goal is the partitioning of resources between sun and shade leaf layers and configuring these leaves with different traits. Sun leaves are grown to minimize carboxylation limitations, while shade leaves are adapted to minimize light limitation. For example, sun leaves often have higher leaf mass per area (LMA) and lower nitrogen (N) concentration, thicker palisade parenchyma tissues, and more mesophyll cells (Björkman, 1981; Anderson et al., 1988; Hikosaka and Terashima, 1996), which combined, supports higher photosynthetic rates on an area basis compared to shade leaves (Evans et al., 1988; Evans and Poorter, 2001; Niinemets and Valladares, 2004; and others). In contrast, shade leaves often have lower LMA and similar N on a mass basis, but a larger proportion of N is allocated to chlorophyll to enhance light capture, compensating for lower irradiance to achieve photosynthetic capacity similar to that of sun leaves (Boardman, 1977; Hikosaka and Terashima, 1996; Poorter et al., 2009). How additional leaf traits differ between sun and shade positions is less well known.

Forest canopy studies demonstrate that sun-shade leaf differences in LMA, N, chlorophylls (the combined value of chlorophyll a and b, as Chl ab), and photosynthetic rates are strongly correlated with the vertical light gradient within tree canopies (Farquhar, 1989; Poorter et al., 1995; Niinemets et al., 1999), supporting the optimal resource partitioning hypothesis to maximize canopy photosynthetic production (Shipley et al., 2006). However, maximum photosynthetic rates are rarely achieved within the canopy (Meir et al., 2002; Lloyd et al., 2010; Dewar et al., 2012). Numerous studies have shown that photosynthesis-trait relationships are constrained by physical limitations of leaf architecture (Sack and Scoffini, 2013; Blonder et al., 2017), whole-plant structure or canopy height (Wright et al., 2007; Brodribb and Feild, 2010; Cavaleri and Oberbauer, 2010), and within canopy temperature and/or humidity (Chazdon et al., 1996; Meinzer, 2003), but little is known about the light response of other foliar chemical traits within canopies.

The natural abundance of δ^13^C (the isotopic ratio of ^13^C/^12^C expressed on a ^0^/_00_ relative to a standard) in leaf tissue is a time-integrated measure of CO_2_ assimilation by the plant has served as a surrogate for water use efficiency (WUE, the ratio of carbon gained to water lost during gas exchange (Farquhar et al., 1989). Foliar δ^13^C is determined by the internal and external concentration of CO_2_ in leaves, and is sensitive to environmental factors influencing stomatal conductance (i.e., water stress (Ehleringer, 1991), internal resistance in high LMA leaves (Cordell et al., 1998), and decreasing partial pressure of CO_2_ with elevation (Körner et al., 1991). Additionally, δ^13^C has been shown to differ between overstory (direct sunlight) and understory plants (Medina and Minchin, 1980; Ehleringer et al., 1986), and in the source air for plants in different positions within the canopy (Sternberg et al., 1989; Buchmann et al., 1996); however, the effect of different light regimes within the same canopies has had little study (Garten and Taylor, 1992; Holtum and Winter, 2005).

Beyond foliar traits involved in light capture and growth, there are a number of additional chemicals known to be functionally important in leaves, and which are predictors of plant adaptation to environmental conditions (Díaz et al., 1998). We group these traits into three additional categories: defense (phenols, tannins, lignin and cellulose), metabolic regulation, macronutrients (phosphorous P, calcium Ca, potassium K, magnesium Mg), and other micronutrients (boron B, iron Fe, manganese Mn, zinc Zn). Polyphenols encompass a wide array of phenolic and tannic compounds (measured here as bulk phenols and condensed tannins), and are synthesized for chemical defense against pest and pathogens, which are particularly abundant in tropical forests, (Coley and Barone, 1996), and are also used for protection against high solar radiation (e.g., anthocyanins), antioxidants, and other foliar protections (Grace, 2005). In addition, lignin and cellulose affect leaf digestibility and toughness as both defense and structural support (Weng and Chapple, 2010). Macronutrients (P, Ca, K, Mg) and micronutrients (B, Fe, Mn, Zn) play key roles in regulating metabolic activities, cellular allocation, and growth. For example, K assists in maintaining stomatal control and osmotic potential necessary for efficient photosynthesis and respiration, while Ca and Mn play roles in generating cell walls and the chloroplast structure (Salisbury and Ross, 1992). Whether these traits differ among sun and shade positions within canopies is not well known.

Understanding how foliar traits vary in sun and shade leaves may be complicated in tropical forest canopies depending upon whether comparisons are made within or between species (Ter Steege et al., 2006; Wright et al., 2010; Asner and Martin, 2016). High species and structural diversity, in addition to multiple environmental filters, can drive trait variation (Messier et al., 2010). Previous studies of foliar chemistry have focused on the effects of soil fertility, elevation and climate on N, P, and base cation (Ca, Mg, K) concentrations, and morphological traits such as LMA and thickness, in upper canopy leaves. For example, global variation in LMA measured across biomes or within humid tropical forests range from 14–1,500 g m^-2^ and 113–446 g m^-2^, respectively (Wright et al., 2004; Asner and Martin, 2016). A wide range in LMA was even measured within one species (*Metrosideros polymorpha* Gaudich.) growing across a range of elevations and substrates in the Hawaiian islands (Martin and Asner, 2009). Furthermore, a recent study along multiple elevation gradients in the western Amazon greatly expanded the portfolio of canopy foliar traits, and integrated the role of interspecific variation in the list of explanatory factors regulating variation in foliar chemistry in upper-canopy, sunlit leaves (Asner et al., 2014b). Given such broad species and environmental variation in sunlit foliar traits, is it possible to determine variation between sun and shade leaves?

Here we assess differences in 19 foliar traits in paired sun and shade leaves along a 3,500-m humid tropical forest elevation gradient in Peru. We quantify trait variation within and between these differing leaf types to understand whole-leaf adaptation to both local light and large-scale climatic (elevation) conditions. We focused on traits that: (i) mediate or are indicative of photosynthesis and carbon uptake (Chl ab, carotenoids and δ^13^C); (ii) are related to structure and chemical defense; and (iii) are related to metabolism including macronutrient and micronutrients. The elevation gradient, combined with the great diversity of canopy species included in our study, affords a means to compare and contrast foliar trait responses in sunlit and shade leaves across a range of forest structural and compositional contexts.

Using this elevation gradient, we ask: Do foliar chemical traits differ between sun and shade leaves, and do genetic and/or environmental filters, such as climate and soils, limit the variation between sun and shade leaf traits? We expect that light-sensitive traits, such as LMA and photosynthetic pigments, will differ between sun and shade leaves, and that this plasticity, associated with decreasing light availability within canopies, may be coupled with adaptive sensitivity to changing environmental conditions such as incoming solar radiation along the elevation gradient. However, we do not know if other foliar traits, such as those related to defense and nonphotosynthetic metabolism, will follow a similar pattern.

## Methods

### Field Sampling

We measured foliar traits from top-of-canopy, fully expanded sun and paired within-canopy shade leaves in 385 tropical trees in ten sites arrayed along an Andes-to-Amazon elevation gradient. This gradient stretches from 200 m elevation in the Amazonian lowlands to 3500 m at the Andean tree line (**Table 1**, **Figure S1**). Changing environmental conditions along this gradient include decreasing temperature with increasing elevation, as well as a U-shaped pattern of incoming solar radiation, with lowest radiation levels in the submontane region (Fyllas et al., 2017; Malhi et al., 2017). Along the gradient, mean annual precipitation (MAP) varies from 1,600–5,300 mm yr^-1^, high enough to classify all sites as moist or wet tropical forest. The 1-ha forest sites were installed by the Andes Biodiversity Ecosystems Research Group (ABERG, http://www.andesconservation.org) and are part of the ForestPlots (https://www.forestplots.net/) and Global Ecosystems Monitoring Network (GEM; http://gem.tropicalforests.ox.ac.uk/projects/aberg) networks. Mean annual temperature (MAT) ranges from 9.0°C at the highest elevation site to 24.4°C at the lowland sites. A comparison of MAT and elevation measured at the individual sites indicate a nearly one-to-one linear relationship (MAT = 25.7-0.005 x Elevation, R^2^ = 0.99, *p* < 0.05), therefore we chose to analyze environmental influences on trait relationships in terms of elevation rather than presume a causal relationship with temperature.

**Table 1:** Site characteristics including soil type, location, elevation, mean annual precipitation (MAP), mean annual temperature (MAT), ambient solar radiation for ten plots sampled for canopy foliar traits along the Andes-Amazon elevation gradient.

Site	Soil Type	Latitude	Longitude	Elevation (m)	MAP (mm yr^-1^)	MAT (°C)	Solar radiation (GJ m^-2^ yr^-1^)
Tambopata; TAM-06	Alisol	−12.8386	−69.2960	215	1900	24.4	4.80
Tambopata; TAM-05	Cambisol	−12.8303	−69.2706	223	1900	24.4	4.80
Pantiacolla; PAN-02	Plintosol	−12.6496	−71.2627	595	2366	23.5	3.82
Pantiacolla; PAN-03	Alisol	−12.6383	−71.2745	848	2835	21.9	3.82
San Pedro; SPD-02	Cambisol	−13.0491	−71.5366	1494	5302	18.8	4.07
San Pedro; SPD-01	Cambisol	−13.0474	−71.5424	1713	5302	17.4	4.35
Trocha Union; TRU-04	Umbrisol	−13.1059	−71.5893	2719	2318	13.5	3.49
Esperanza; ESP-01	Umbrisol	−13.1759	−71.5948	2868	1560	13.1	3.51
Wayquecha; WAY-01	Cambisol	−13.1907	−71.5875	3045	1560	11.8	3.51
Acjanaco; ACJ-01	Cambisol	−13.1469	−71.6323	3537	1980	9.0	4.23

Soils at sites above 1,500 m are classified in the FAO soil system as Cambisols. The two lowland sites (<500 m above sea level), are a Cambisol located on *terra firme* clay substrate of late Pleistocene age (TAM-05) and an Alisol of very low nutrient concentration on an inactive high-fertility floodplain of late Holocene age (TAM-06; Quesada et al., 2009). The two submontane sites (500–1,000 m elevation) are located on a highly weathered, mineral rich Alisol soil (PAN-03) and a weathered, clay-rich Plinthosol (PAN-02) on the Pantiacolla front range of the Andes, both soils supporting lower nutrient concentrations compared to higher fertility Cambisols. The highest elevation site, ACJ-01, is located at treeline on extremely thin soils. This site is steeply sloping and the plant community is dominated by individuals in the genus Melastomataceae, indicating very poor nutrient soils.

Foliar sampling was undertaken between April and November 2013 as part of the CHAMBASA (Challenging Attempt to Measure Biotic Attributes along the Slopes of the Andes) project. Based on the most recently available census and diameter data for each plot, a sampling protocol was adopted wherein species were sampled that maximally contributed to plot basal area (a proxy for plot biomass or crown area). We aimed to collect the minimum number of species that contributed to 80% of basal area; however, in the diverse lowland forest plots, we only sampled species comprising 60%–70% of plot basal area. For each selected species, 3–5 individual trees were chosen for sampling (five trees in submontane and montane plots; three trees in lowland plots). If three trees were not available in the plot, we sampled additional individuals of the same species from an area immediately surrounding the plot. The collected samples were comprised of 134 species from 89 genera in 49 families. At a given site, between 9 and 26 unique species were sampled (**Table S1**).

Leaf collections were conducted using tree-climbing techniques to ensure that mature leaf samples were collected from accurate sun and shade locations within each canopy. Sun leaves are considered leaves found on the outermost layer of the canopy and are exposed to full sunlight at least 80% of daylight hours. If multiple layers were present in the canopy, leaves were collected from the lowest suitable layer and were designated as shade leaves. Relative light levels at these locations were usually < 10% of top of canopy but precise light measurements are still forth coming (Shenkin *pers comm*). Once acquired, each sample was immediately packed in plastic bag and stored on ice in the dark until being transported to a local site for processing within 30 min following collection. Samples were cryo-cooled or dried on site immediately after measurements of fresh weight and leaf area were made. A foliar profile, including 18 chemical traits and LMA, was developed for each sample, sun and shade.

### Laboratory Assays

Branches (generally 1–2 m in size with multiple branchlets) of mature leaves were sealed in polyethylene bags in the field to maintain moisture, stored on ice in coolers, and transported to a local site for processing within 3 h. A subset of leaves was selected from the branches for scanning and weighing. Leaf area was determined on a 600 dots-per-inch flatbed top-illumination optical scanner, using enough leaves to fill one scan area of 21 cm × 25 cm (up to about 35 leaves per sample depending on leaf size). Petioles were removed from each leaf prior to scanning, and midveins were removed when they exceeded 1 mm in diameter. Leaves exceeding the surface area of the scanner were cut into sections until 1–2 full scan areas were completed. The scanned leaves were dried at 70°C for 72 h before dry mass (DM) was measured. LMA was calculated as g DM m^-2^. From a subset of leaves, leaf discs (at least 30 per leaf) were immediately taken from approximately 6–12 randomly selected leaves and transferred to −80°C cryogenic shipping containers. The remaining leaves were detached from the branches and subsamples were selected to represent the range of colors and conditions found among all leaves collected from the branches (such as leaf size or slight variation in age within mature leaves). When epiphylls were encountered, they were removed, along with dust and debris, prior to drying in mobile ovens at 70°C for 72 h followed by vacuum sealing for transport.

Detailed chemical analysis protocols, along with instrument and standards information are downloadable from the Spectranomics Program website (https://gao.asu.edu/spectranomics-protocols), and are summarized here. Dried foliage was ground in a 20-mesh Wiley mill, and subsets were analyzed for a variety of elements and carbon fractions. Total element concentration of macro- (P, Ca, K, Mg) and micronutrients (B, Fe, Mn, Zn) were determined in 0.4 g dry leaf tissue by inductively coupled plasma spectroscopy (ICP-OES; Therma Jarrel-Ash, Waltham MA) after microwave digestion in 10 ml concentrated (~70%) nitric acid solution (CEM MARSXpress; Matthews NC). One blank and two reference standards (Peach NIST SRM 1547 and internal lemon leaf) were digested and measured with each set of 40 foliar samples to track the reproducibility and accuracy of the method.

Carbon fractions including nonstructural carbohydrates (NSC), cellulose and lignin were determined in 0.5 g dry ground leaf tissue through using sequential digestion of increasing acidity in a fiber analyzer (Ankom Technology, Macedon NY). Carbon fractions are presented on an ash-free DM basis following ignition of the remaining sample at 500°C for 5.5 h. A lemon leaf standard was used as a reference with each run to ensure consistency across runs. A subset of the ground material was further processed to a fine powder for determination of total C and N concentration by combustion-reduction elemental analysis (Costec Analytical Technologies Inc. Valencia, CA). Following combustion, a portion of the gas is routed through a mass spectrometer (Picarro Inc. Santa Clara, CA, USA) where the separate isotopes of C^12^ and C^13^ are measured. The isotopic ratio δ^13^C is calculated against a reference standard as

δ13C(000)=[((C13/C12sample)/(C13/C12standard))−1]×1000.

Frozen leaf disks were used for the chl ab, carotenoid, phenol and tannin determinations. For phenols and tannins, disks were ground in 95% methanol on the high throughput tissue homogenizer. A portion of the solution was further diluted and incubated on an orbital shaker at room temperature (15°C–18°C) in the dark for 48 h to ensure proper phenol extraction (Ainsworth and Gillespie, 2007). A second portion of the solution was further diluted in a 2-ml centrifuge tube containing 10 mg Polyvinylpyrrolidone (PVP) and incubated on ice for 30 min after vortexing. Following centrifugation, 75% of the supernatant was placed in a new centrifuge tube containing another 10 mg PVP for a second precipitation step (Toth and Pavia, 2001). The total phenolic concentration in solution was determined colorimetrically using the Folin-Ciocalteau method. Phenol concentrations were measured in Gallic Acid Equivalents (GAE) relative to an eight-point Gallic acid standard curve. Chlorophyll (chl ab) and carotenoid concentrations were quantified using two frozen leaf disks (total area 1.54 cm^2^). These disks were rapidly ground in 1.5 ml centrifuge tubes containing 0.75 ml 100% acetone on a high throughput tissue homogenizer (Troemner, Thorofare, NJ) with a small amount of MgCO_3_ to prevent acidification. Following dilution and centrifugation for 3 min at 3,000 rpm, the absorbance of the supernatant was measured using a dual-beam scanning UV-VIS spectrometer (Lambda 25, Perkin Elmer, Beaconsfield, UK).

### Statistical Analyses

Because sun and shade leaves were collected across sites differing in elevation, climate (i.e. MAP, MAT) and geology (**Table 1**), canopy position at the tree level was effectively nested within site and could not be compared in a fully randomized way; therefore, nested analyses of variance (ANOVA) was used to first define sources of variation in canopy position and site effects in each of the foliar traits to determine if sun leaves differed from shade leaves within canopies at each site. Mass-based foliar traits at the tree level including LMA, N, Chl ab, carotenoids, P, K, and Mg were log_10_-transformed to meet the assumptions of normality. Ca was transformed by taking the square root to account for the large number of near-zero values. Mass-based foliar traits were converted to area units by dividing by LMA for analysis. For analyses at the individual tree level, all area-based measures except chlorophylls, carotenoids and δ^13^C were log_10_-transformed to meet the assumptions of normality. For analyses across sites at the landscape-scale, foliar traits were averaged by site and canopy position. We used ordinary least squares regression to assess relationships between site-level mean values for each canopy trait between sun and shade canopy layers, elevation and their interaction. If there was no interaction between site and canopy position, relationships among sun or shade leaves and elevation were calculated using linear least squares regression, and the offset between sun and shade position was determined with matched pairs t-tests for each trait.

With the goal of examining how the magnitude of variation in chemical traits among sun or shade leaves is distributed within and across species, we assessed within tree variation as well as intraspecific and interspecific coefficients of variation (CV) calculated with untransformed data regressed against elevation. The magnitude of variation among sun and shade leaves within trees at each site was calculated as the CV of the standardized difference between sun and shade leaves within the trees at each site. Intraspecific CV including sun and shade leaves was calculated for each species within a site as the standard deviation in the trait value divided by the mean trait value. The mean of the intraspecific CV including sun and shade leaves values was then used for the analysis. Interspecific CV was calculated as the standard deviation across the species mean values for sun or shade leaves standardized by the mean trait value.

In addition, to determine how the total variance is distributed among taxonomic grouping, canopy position, and site, we developed nested ANOVA models with random effects using the Residual Maximum Likelihood method using SAS JMP 10.0 statistical software package (SAS Institute Inc. Cary NC). For intraspecific and interspecific variation we included the taxonomic levels of genus (g), and species nested within genus (s), as well as canopy position (P) and landscape-level environmental components incorporated as site (T). All effects were treated as random. In each model, *y* is any chemical trait for each canopy sample. This value was modeled as the sum of the mean value for the entire dataset *µ*, the nested genetic effects (genus *j*, and species *i* within genus *j*), the canopy position (P) nested within genus and species, all within the site effect (T; sensu Fyllas et al., 2009; Messier et al., 2010), and the residual error of the measurement *e*:

$$y={µ}_{}+\;{\text{g}}_{jl}+\;s_{ijl}+\;{\text{P}}_{kijl}+\;{\text{T}}_{l}+e_{ijkl}$$

The total variance about the mean for a given trait was therefore quantitatively parsed into the variance explained by genera ($\sigma_{g}^{2}$), species within genera ($\sigma_{s}^{2}$), canopy position ($\sigma_{P}^{2}$), site ($\sigma_{T}^{2}$), and specimens within species ($\sigma_{e}^{2}$):

$$\sigma_{\text{total}}^{2}=\sigma_{g}^{2}+\sigma_{s}^{2}+\sigma_{P}^{2}+\sigma_{T}^{2}+\sigma_{e}^{2}.$$

If in a given model, the last term ($\sigma_{e}^{2}$) accounted for a high percentage of the total variance, then we concluded that site characteristics and taxonomy did not explain the data well. We refer to this as the model residual.

One limitation of this analysis is that it describes the overall variation explained by each input variable. We acknowledge that not all taxa have equal variance; some may have tightly clumped chemical signatures whereas others may vary widely. This analysis will not pick up such trends. Instead, the method quantifies the entire pattern of taxonomic grouping or lack thereof relative to canopy position and site and residual effects. Previous work successfully tested the validity of nested random effects modeling for analysis of taxonomic partitioning of foliar chemical traits (Fyllas et al., 2009; Asner et al., 2014b) but has not determined if there is an effect of shade leaves beyond light sensitive traits such as LMA or leaf dry matter content (Messier et al., 2010).

## Results

### Growth Traits

**Figure 1:**
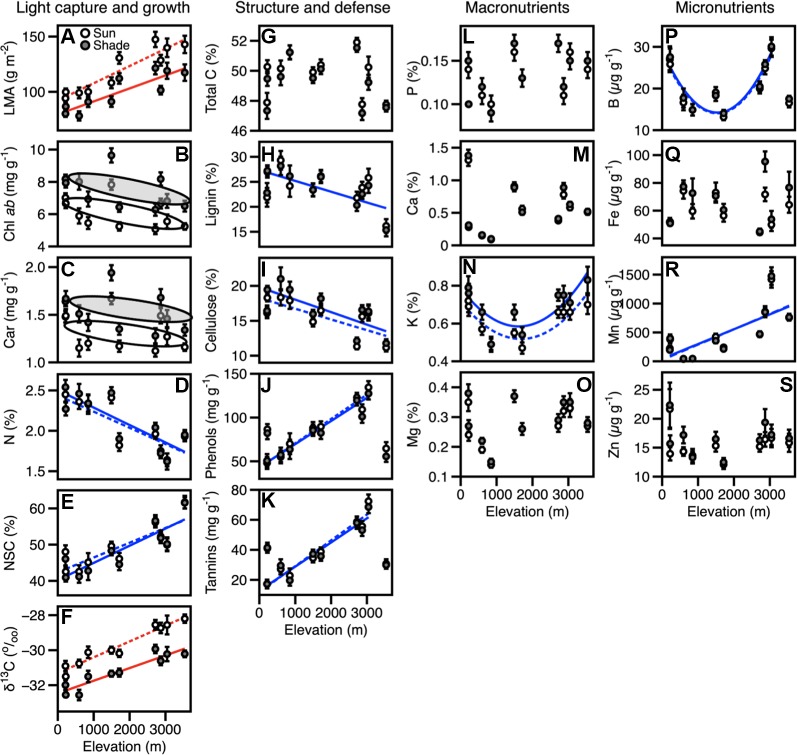
Site mean values for foliar traits, expressed on a mass-basis in sun (open circles) and shade (closed circles) along an elevation gradient are shown. **(A–S)** Variation in foliar traits corresponding to sun-shade canopy positions along an Andes-Amazon elevation gradient in Peru. Error bars represent standard errors. Solid and dotted lines connect trait values across the sites for shade and sun leaves respectively. Red lines indicate significant relationships with elevation. Blue lines show significant trends with elevation for the values of sun and shade when these trait values did not differ between canopy positions. Grey and white ovals indicate significant differences in traits between sun and shade leaves that are not correlated with elevation. Number of individuals per site are given in **Table S1**. Equations for trait-elevation relationships are given in **Table S4**.

Foliar traits displayed a high degree of variation among 385 tropical tree canopies in the 10 sites along the elevation gradient (**Figure 1**, **Table S2**), including some differences between sun and shade leaves within each crown. We found that LMA and δ^13^C concentrations in sun and shade leaves increased with elevation (**Figures 1A, F**, **Tables 2**, **3**). Mean LMA increased 64% from the lowest- to highest-elevation site while maintaining a near-constant offset in LMA of 19.2 g m^-2^ between sun and shade leaves (**Figure 1A**, **Table 3**). Foliar δ^13^C was 1.42^0^/_00_ less negative in sun than shade leaves, while monotonically increasing by 100% across the length of the gradient (**Figure 1G**, **Tables 2**, **3**). Differences in these traits were consistent across elevation therefore elevation-based relationships for LMA and δ^13^C were determined separately for sun and shade canopy positions (**Table 2**). Chlorophyll ab (Chl ab) and carotenoid (Car) concentrations averaged 1.4 and 0.2 mg g^-1^ higher in shade compared to sun leaves (**Figures 1B, C**, **Table 3**), but did not vary systematically with elevation. Mean foliar N (mass %) decreased substantially with increasing elevation in the communities along the gradient, but were not different between sun and shade leaves (**Figure 1D**, **Table 3**). When converted to an area-basis (**Tables S3****–****S5**), dividing by LMA, N, NSC, and δ^13^C concentrations differed between sun and shade leaves, while Chl ab and carotenoid concentrations were similar in both canopy positions (**Tables S3****–****S5**). Photosynthetic pigments and NSC displayed positive relationships with elevation on an area-basis whereas δ^13^C decreased with increasing elevation and N did not vary consistently across elevation (**Tables S3****–****S5**).

**Table 2:** Relationships between site-level mean leaf traits on a mass basis from the sun or shade layer of the canopy and elevation.

Trait	Sun layer		Shade layer	
	R^2^	Equation	R^2^	Equation
***Light capture and growth***				
LMA	0.87 (7.9)***	15.6 x Elevation + 92.1	0.78 (8.3)***	11.8 x Elevation + 79.6
N	0.68 (0.2)**	−0.2 x Elevation + 2.4	0.67 (0.2)**	−0.2 x Elevation + 2.5
Chlorophyll *ab*	NS		NS	
Carotenoids	NS		NS	
NSC	0.70 (3.5)**	4.1 x Elevation + 42.4	0.76 (3.6)**	4.7 x Elevation + 40.2
δ^13^C	0.95 (0.3)***	0.9 x Elevation + −31.3	0.88 (0.4)***	0.7 x Elevation + −32.5
***Structure and defense***				
C	NS		NS	
Lignin	NS		0.51 (2.8)*	−2.2 x Elevation + 27.3
Cellulose	0.55 (1.8)*	−1.5 x Elevation + 18.3	0.54 (2.2)*	−1.8 x Elevation + 19.8
Phenols^1^	0.96 (7.0)***	27.8 x Elevation + 42.7	0.93 (7.8)***	25.1 x Elevation + 43.6
Tannins^1^	0.94 (5.1)***	17.2 x Elevation + 11.9	0.92 (5.8)***	16.1 x Elevation + 12.7
***Macronutrients***				
P	NS		NS	
Ca	NS		NS	
K^1^	0.69 (0.06)**	0.07*elev^2^-0.24*elev+0.72	0.61 (0.08)**	0.08*elev^2^-0.25elev+0.79
Mg	NS		NS	
***Micronutrients***				
B^1^	0.79 (3.25)**	6.2*elev^2^-18.9*elev+28.5	0.84 (2.82)**	6.4*elev^2^-19.6*elev+29.3
Fe	NS		NS	
Mn	0.57 (295.1)*	257.1 x Elevation + 41.7	0.56 (312.0)*	265.3 x Elevation + 24.1
Zn	NS		NS	

Asterisks indicate significant levels as *p < 0.001, **p < 0.01, and ***p < 0.05. ^1^ or NS (nonsignificant) sites ACJ-01 and TAM-05 excluded from regressions due to unusual soil properties interacting with these traits. Elev is elevation. See methods for details.

Correlation value (R^2^) is provided with root-mean-squared error (RMSE) in parentheses. The equations are reported relative to elevation in km. LMA is leaf mass per area. NSC are nonstructural carbohydrates.

**Table 3:** Results of nested ANOVA testing for differences among leaf traits on a mass-basis between sun and shade leaves and site^1^.

Response variable	Source of variation				
	Site		Canopy position (site)		Offset
	F	*P*	F	*P*	µ ± STERR
***Light capture and growth***					
**LMA**	22.97	<0.01	7.70	<0.01	−19.18 ± 1.04*
**N**	31.63	<0.01	0.30	NS	
**Chlorophyll *ab***	19.26	<0.01	8.33	<0.01	1.43 ± 0.10*
**Carotenoids**	19.01	<0.01	4.35	<0.01	0.21 ± 0.02*
**Soluble C**	30.90	<0.01	0.35	NS	
**δ^13^C**	35.22	<0.01	16.56	<0.01	−1.42 ± 0.06*
***Structure and defense***					
**Total C**	23.98	<0.01	0.75	NS	
**Lignin**	11.34	<0.01	0.26	NS	
**Cellulose**	28.65	<0.01	1.46	NS	
**Phenols**	24.58	<0.01	0.30	NS	
**Tannins**	37.85	<0.01	0.20	NS	
***Macronutrients***					
**P**	36.95	<0.01	0.35	NS	
**Ca**	132.97	<0.01	0.46	NS	
**K**	11.93	<0.01	1.70	NS	
**Mg**	25.31	<0.01	0.61	NS	
***Micronutrients***					
**B**	23.99	<0.01	0.24	NS	
**Fe**	23.39	<0.01	1.63	NS	
**Mn**	68.02	<0.01	0.08	NS	
**Zn**	5.01	<0.01	0.52	NS	

^1^Sample collection at sites varying in elevation, light environment, and geology meant that canopy position was effectively nested within site.

LMA, leaf mass per unit area; LMA, N, chlorophyll ab, carotenoids, P, K, and Mg were log-transformed before analysis. Ca was transformed by square root.

STERR is standard error.

* indicates significant offset at p < 0.01. NS is nonsignificant.

Mean offset among canopy position (shade to sun) across all sites is also shown (Matched pairs t-test, p <0.001). LMA is leaf mass per area. NSC are nonstructural carbohydrates.

### Other Traits

Foliar carbon components NSC (lignin, cellulose, and total C) and defense traits were similar in sun and shade leaves, but differed relative to elevation. Total C was invariant with respect to elevation, while lignin and cellulose decreased with elevation (**Figures 1G–I**, **Table S2**). On the other hand, foliar NSC increased with increasing elevation (**Figure 1E**). In contrast, phenols and tannins increased with elevation up 3,045 m (WAY-01), where average concentrations were 130 mg g^-1^ and 70 mg g^-1^, respectively, after which concentrations of these defense compounds decreased by approximately 50% at the highest elevation site (AJC-01). Phenols and tannins were significantly correlated with elevation only when the extremely nutrient-poor sites (TAM-05 and ACJ-01) were not included in the relationship (**Figures 1J–K**).

Wide ranging values in macronutrients and micronutrients within crowns and among canopies contributed to a lack of distinction between sun and shade leaves, and limited elevation-dependent trends with a few exceptions (**Figures 1L–S**). Mn concentrations increased from 45 μg g^-1^ to almost 1500 μg g^-1^ up to 3,000 m, but decreased by nearly 30% to 850 μg g^-1^ at the highest elevation site (**Figure 1R**). Differing from other traits, K and B displayed a U-shaped pattern, with higher values in the lowlands and montane sites (0.66%–0.72% and 26–30 μg g^-1^, respectively; **Figures 1N, P**), and substantially lower values in the submontane sites (0.47%–0.54% and 13–14 μg g^-1^). Different from K, foliar concentrations of B were much lower at the highest elevation site (ACJ-01; 16–17 μg g^-1^).

Expressed on an area-basis, only total C, lignin, and P concentrations were higher in sun compared to shade leaves and these offsets were maintained across the elevation gradient (**Tables S3****–****S5**). Additionally, nearly all foliar traits beyond growth related traits showed significant trends with elevation when expressed on an area-basis (**Table S5**). The exceptions were lignin, cellulose, and Ca.

### Sources of Trait Variation

Multiple canopy foliar traits exhibited variation associated with elevation; however, only growth-related traits differed between sun and shade leaves. On a mass basis, canopy position accounted for a significant portion of the variation among the sites for the light-sensitive traits including Chl ab, carotenoids and δ^13^C, as well as LMA (**Table 3**). Chl ab and Car were 19% and 13% higher, respectively, in shade than in sun leaves (**Tables 3**, **S2**; t = 13.9 and 10.8 respectively; *p* < 0.001). Foliar concentrations of δ^13^C were 4.5% less negative in shade than sun foliage (t = -22.4, *p* < 0.001). LMA was 19% higher (t = −18.5; *p* < 0.001) in sun than in shade leaves, resulting in similar values for photosynthetic pigments (Chl ab, Car) in sun and shade leaves when concentrations were calculated on a leaf-area basis (**Tables S3**, **S4**). Higher LMA in sun leaves also resulted in significantly higher area-based N, soluble C, δ^13^C, P, and lignin, in sun compared to shade leaves (**Tables S3**, **S4**). Because the differences between sun and shade leaves calculated on an area basis are almost entirely due to the changes in LMA (Lloyd et al., 2013), we focus most of our remaining analyses on mass-based traits. There was substantial variation in macronutrient and micronutrient concentrations within and among some sites, but these traits did not show sun-shade differences of sufficient magnitude to separate them on a mass or area basis (**Tables 3**, **S2****–****S5**).

**Figure 2:**
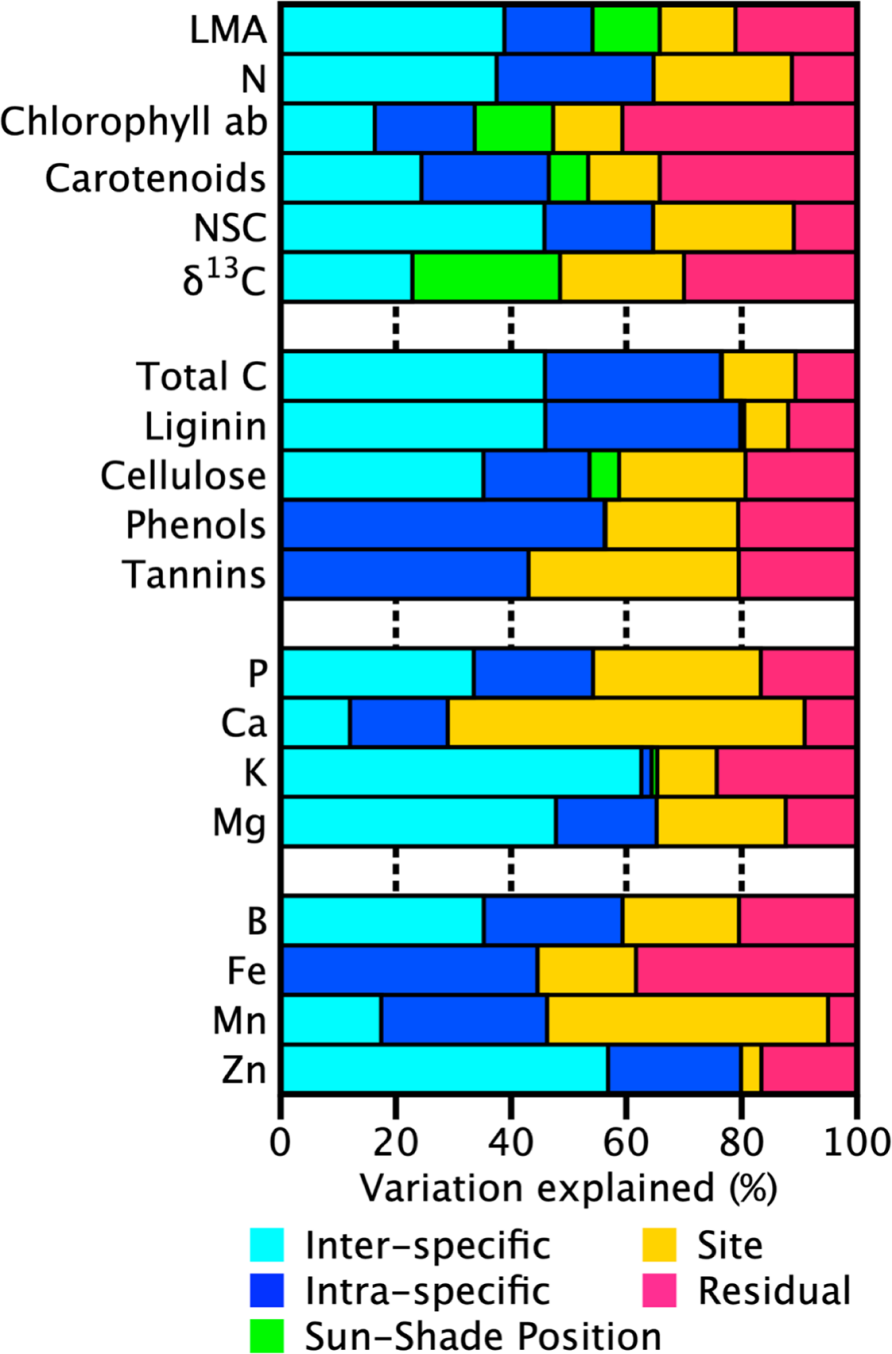
Partitioning of the variance for each tree canopy chemical trait into site, canopy position (sun or shade), phylogenetic variation, and unexplained residual variation for 10 sites along an Andean-Amazon gradient in Peru. The site component incorporates environmental variation in geology, topography, elevation, temperature, precipitation, radiation. Here, phylogenetic variation is separated into intraspecific and interspecific components (species and genera + family partitions respectively). Unexplained residuals are comprised of measurement error and other nonsite-related sources of uncertainty such as tree and foliage selection.

The high degree of species turnover along this elevation gradient makes disentangling taxonomic versus site effects difficult, and with only one plot per site, this was not the focus of this study. We used a nested approach to partition the variation to determine if, once other sources of variation are accounted for, if canopy position might emerge as an important factor. Analysis of the partitioning of the trait variation by canopy position (sun or shade), site (MAT, MAP, geology, topography, elevation), genetic (intraspecific and interspecific), and residual (measurement error and other nonsite related sources) components, indicated that canopy position figured into the measured variation in LMA, Chl ab, and Car (12%, 14%, and 7%, respectively, **Figure 2**). Also, a larger proportion of the variance in foliar δ^13^C (25%) was explained by canopy position than any other factor other than the model residual. On the other hand, canopy position accounted for less than 5% of the variance in other traits. Concentrations of photosynthetic pigments (Chl ab and carotenoids) in sun and shade leaves were similar on an area-basis, compensated for the most part by changes in LMA.

As has been found previously (Asner et al., 2015; Asner et al., 2017), genetics (intraspecific/interspecific) explained at least 50% of the variation in most leaf traits (**Figure 2**). However, the degree of partitioning between intraspecific and interspecific components of variation was not consistent among the foliar traits. For many traits the variance attributed to intraspecific or interspecific differences was less than 15%, but interspecific variation was more than 20% higher than intraspecific variation for some nutrients (K, Mg, B, and Zn). In contrast, intraspecific variation was higher in phenols and Fe, 19% and 24% respectively. LMA, δ^13^C and total C showed almost no difference (<5%) in the variance attributed to intraspecific or interspecific variation. Site explained 22%–62% of the variation in most macronutrient and micronutrient as well as defense compounds (phenols, tannins). A large portion of the total variation in Mn was also explained by site (49%). However, less than 10% of the total variation in K, lignin, and Zn was attributable to site. Variation in these traits was dominated by intra and interspecific variation (65%–80%).

**Figure 3:**
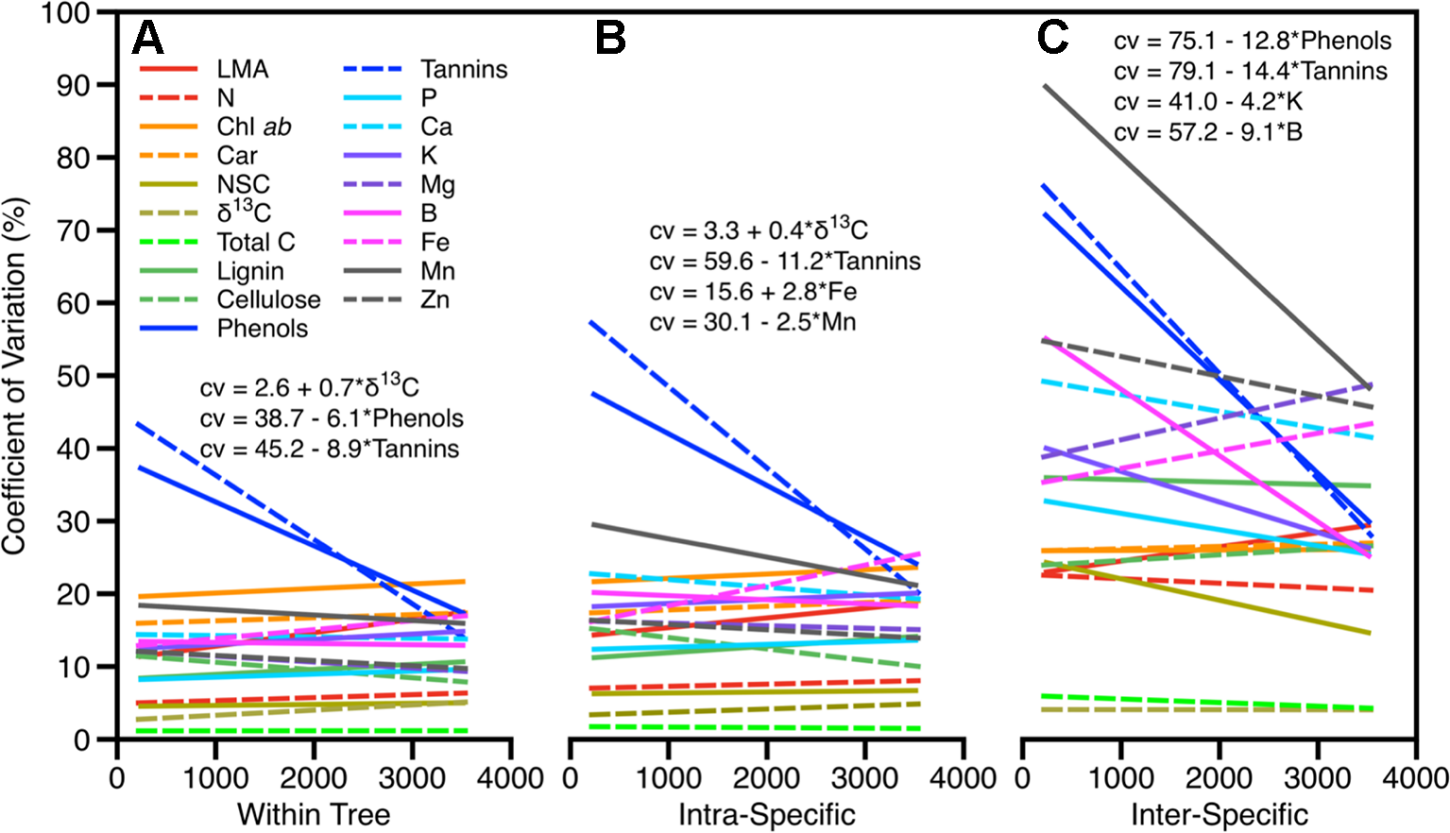
Degree of variation in foliar traits within all sites as they vary across the elevation gradient for **(A)** within trees due to canopy position (sun and shade), **(B)** within species, and **(C)** among species. Significant relationships (*p* < 0.05) between trait variation and elevation are given.

The degree of trait variation between sun and shade leaves was less than or similar to the variation within species at a site and did not change with elevation across the gradient with the exception of defense chemicals (phenols and tannins) and δ^13^C concentration (**Figure 3**). Variation in these defensive traits was highest among all traits, ranging between 17% and 80%, and was negatively correlated with elevation in canopy position and within and across species. Variation in δ^13^C concentration also changed along the elevation gradient but the magnitude of change was relatively low: 25% among species and 15% within species. Variation in δ^13^C within species and between sun and shade leaves increased slightly with increasing elevation (**Figure 3**, **Table S6**). Variation in macronutrients and micronutrients was similar to other foliar traits (6%–21%, 9%–30%, and 21%–90% within tree, intraspecific, and interspecific respectively). A small number of these were significantly correlated with elevation; however, there was no systematic pattern (**Figure 3**). Intraspecific variations in Fe and Mn concentrations were positively correlated with elevation, while intraspecific variation in K and B decreased with increasing elevation.

## Discussion

We found that taxonomic and environmental controls on variation in shade leaf trait patterns mirror or match the variation observed in sun leaves. For most traits (i.e. N, foliar nutrients or defense compounds), there was no significant difference between sun and shade leaves. Significant within-canopy differences between sun and shade leaves were measured among certain growth-related traits including LMA, photosynthetic pigments, and δ^13^C (**Figure 1**, **Tables 2**, **3**), but these traits maintain constant offsets, suggesting characteristics of shade leaves can be derived from those measured in sun leaves. In this discussion we first review data from the few studies examining foliar traits in sun and shade leaves that are not directly associated with photosynthesis. We then discuss the response of sun and shade leaves to environmental variation and compositional turnover along this elevation gradient. We conclude by explaining how this study benefits landscape-scale remote sensing.

Our observed lack of sun-shade differentiation in foliar chemical traits beyond those related to photosynthesis and growth is poorly studied in tropical evergreen species. Grubb (1977) found little difference between sun and shade leaves for C, Fe, N, P and slightly higher concentrations of K and Mg in shade leaves in a number of tropical forests. This was also shown more recently for N and P in a large number of species in moist (Poorter and Rozendaal, 2008) and dry tropical forests (Markesteijn et al., 2007). Foliar concentrations of phenols and NSC were lower, and lignin was higher, in shade leaves of gymnosperms and deciduous trees among a number of species (Poorter et al., 2006). In conifer needles, N, P, K and cellulose concentrations were higher in shade leaves, while Ca concentrations were lower (Richardson, 2004).

The role of compositional turnover in driving changes in foliar traits of tree species in the ten sites along this elevation gradient was presented in Asner et al. (2017) and generally follows patterns on elevation gradients found globally (Asner and Martin, 2016). To what extent shade leaves might adhere to the same pattern was not previously explored. Growth-related traits are known to adapt to their light environment and vary in relation light extinction within canopies. The consistency with which sun-shade trait differences are maintained over the large environmental and compositional gradient sampled here might be surprising. However, light levels of the shade leaves were generally less than 10% of the ambient solar radiation at all sites, likely setting a consistent low light limit for foliar adjustment. The consistent offset between sun and shade leaves in 134 species across varying environmental conditions suggests the plasticity in these traits may be genetically coordinated with maximum low light modification set relative to high light and vice versa.

Our findings show that the portion of light-sensitive trait variability attributed to sun-shade position ranged from 7%–25% with the remaining variation split between taxonomic, site, and residual components (**Figure 2**). The proportions changed only minimally if analyses were done without taxonomic and canopy position nested within a site, indicating the convolved effect of species turnover and site along this gradient. Like past studies, our findings indicate that variation in sunlit canopy foliar traits are controlled primarily by changes in community composition, and secondarily by environmental factors, elevation and substrate (Fyllas et al., 2009; Asner et al., 2014a; Blonder et al., 2017). Variation among the other leaf traits followed patterns previously found, with approximately 50% of the variation was found in the taxonomic fraction (intraplus interspecific), and the remainder split between site and residual effects (**Figure 2**). Moreover, interspecific variation was up to three times higher than intraspecific variation, and was generally constant along the elevation gradient, pointing to the dominating role of species turnover along the elevation gradient (**Figure 3**). The large portion of variation in macronutrients and micronutrients found in the site and residual components, coupled with very low values in these chemical traits measured in the highest elevation site (ACJ-01), are likely related to the overall dystrophic soil conditions throughout the elevation gradient.

As is well documented, leaf structural changes represented by LMA were inversely related to foliar N in sun and shade leaves when calculated on an area basis, but concentrations of photosynthetic pigments converged in value on an area basis (**Tables S3**, **S4**). This finding supports our understanding of increasing leaf area in shaded foliage to increase light interception and photosynthetic capacity at lower subcanopy light intensities. These LMA decreases generally come with increased foliar N content per unit area (Chen et al., 1993; Niinemets, 2007; and many others), which is positively related to maximum photosynthetic rate on an area basis throughout the canopy.

LMA differences (16%) measured among multiple species and a large environmental gradient were far lower than the > 60% recently reported for tropical tree leaves by Keenan and Niinemets (2016) after they scaled traits to a uniform light intensity for comparison. They also reported a 40% difference in mass-based N, where we found none. Whether plasticity between sun and shade leaves of 15%–16% in LMA, and thus area-based N, has a significant effect on canopy photosynthetic capacity in these trees in under investigation (Bentley, L. *pers comm*); however, differences in the δ^13^C concentrations between sun and shade leaves may provide insight into foliar function. Lower δ^13^C values in shade leaves is thought to be related to decreased stomatal resistance in thinner, lower-LMA leaves and/or to subcanopy environments that are cooler and more humid, promoting stomatal opening and enhanced rates of CO_2_ uptake (Niinemets and Valladares, 2004). Both of these conditions should boost CO_2_ uptake and reduce photosynthetic limitations. Lower δ^13^C values from soil respiration may also contribute to lower δ^13^C values in leaves at lower canopy levels (Medina and Minchin, 1980; Sternberg et al., 1989).

## Conclusion

We found significant differences in light-sensitive traits between sun and leaves in 385 canopies of 189 species of tropical rainforest trees. These offsets were maintained across a wide variety of environmental conditions along a 3,500-m elevation gradient suggesting this plasticity associated with light availability is an adaptive change. In contrast, we did not find sun-shade differences in 15 other foliar traits related to defense and metabolism.

These findings of parallel patterns, whether as constant offset or close similarity between many of the canopy sun and shade leaf traits, bear on the effort to scale leaf measurements to landscape and regional levels. For example, ongoing work to map canopy traits using optical remote sensing, particularly imaging spectroscopy, has yielded an understanding of sunlit foliar trait responses to soil fertility, climate, and topography (Ustin et al., 2004; Kokaly et al., 2009; Asner et al., 2011; Asner et al., 2016). Such mapping of sunlit canopy traits has been accomplished over millions of hectares of temperate and tropical forest. However, the relation between what can be mapped at the upper portion of the canopy, also known as the top-of-canopy or canopy skin, and the subcanopy or shaded foliage has left remote sensing with uncertain connection to the remainder of the mapped forest. We found that photosynthesis-related traits such as N, LMA, pigments and δ^13^C, exhibited constant offsets across environmental conditions enabling them to be mapped to upper canopy traits based on principles of light extinction. These principles are well known, and can be applied using other remote sensing techniques that are sensitive to leaf area index and other canopy foliar volumetric properties (Ollinger, 2011). This is not true for shaded leaf traits that are not directly linked to photosynthesis, including numerous macronutrients and defense compounds. However, all of these were of similar magnitude and varied in parallel to their counterparts in sunlit canopy positions. This indicates that top-of-canopy remotely sensed measurements of multiple key foliar chemical traits link directly to whole-canopy foliar properties, including shaded leaves that cannot be directly observed from above. This information is timely because it provides evidence that the rapidly growing area of spectral remote sensing can represent both upper- and lower-canopy foliage, which has been a missing link that can facilitate more robust estimates of canopy function from airborne and satellite platforms.

## Data Availability Statement

The datasets generated and analyzed for this study can be found in the ForestPlots (https://www.forestplots.net/) and Global Ecosystems Monitoring Network (GEM; http://gem.tropicalforests.ox.ac.uk/projects/aberg) networks.

## Author Contributions

The study was conceived and designed by GA, BE, SD, and YM. Field data were collected by RM, LB, AS, NS, KQH, MMP, FCA, and YM. RM carried out laboratory assays. Data analysis was performed by RM. The manuscript was written by RM with contributions from GA, LB, AS, BE, and YM.

## Funding

The field campaign was funded by grants to YM from the UK Natural Environment Research Council (Grant NE/J023418/1), with additional support from European Research Council advanced investigator grants GEM-TRAITS (321131) and T-FORCES (291585) under the European Union's Seventh Framework Programme (FP7/2007-2013). GA and the Spectranomics team were supported by grants from the John D. and Catherine T. MacArthur Foundation and the National Science Foundation (DEB-1146206).

## Conflict of Interest

The authors declare that the research was conducted in the absence of any commercial or financial relationships that could be construed as a potential conflict of interest.
