# Supplementary material for: Covariance of Sun and Shade Leaf Traits Along a Tropical Forest Elevation Gradient
Source: Front Plant Sci. 2020 Jan 31;10:1810. doi: 10.3389/fpls.2019.01810 (PMC7006543; doi:10.3389/fpls.2019.01810)
Supplement: Supplementary file 1 [file Table_1.pdf]

Supplementary Material

1 Supplementary: Covariance of Sun and Shade Leaf Traits Along a Tropical Forest Elevation Gradient

Roberta E. Martin[1,2*], Gregory P. Asner[1,2], Lisa Patrick Bentley[3], Alexander Shenkin[4], Norma Salinas[4,5], Katherine Quispe Huaypar[6], Milenka Montoya Pillco[6], Flor Delis Ccori Álvarez[6], Brian J. Enquist[8,9], Sandra Diaz[7], Yadvinder Malhi[4]

Supporting information: Figures 1; Tables 6.

Figure S1. Map of field site locations along an Andes-to-Amazon elevation gradient in Peru.
Table S1. Summary of number of individuals sampled and the taxonomic composition of the population are given.
Table S2. Mean and standard error values for 18 canopy foliar traits on a mass-basis, LMA (leaf mass per unit area) in sun (bold) and shade leaves from 10 forest sites along an Andes-Amazon elevation gradient in Peru.
Table S3. Mean and standard error values for 18 canopy foliar traits on an area-basis and SLA (specific leaf area; 1/LMA) in sun (bold) and shade leaves from 10 forest sites along an Andes-Amazon elevation gradient in Peru.
Table S4. Results of nested ANOVA testing for differences among leaf traits on an area-basis between sun and shade leaves and site. Mean offset among canopy position across all sites is also shown.
Table S5. Relationships between site-level mean leaf traits on an area basis from the sun or shade layer of the canopy and elevation.
Table S6. Sources of variation in mass-based foliar traits within each site are listed in order of within tree, intra-specific and inter-specific variation for 19 canopy foliar traits from 10 forest sites along an Andes-Amazon elevation gradient in Peru.

Figure S1. Map of field site locations along an Andes-to-Amazon elevation gradient in Peru.

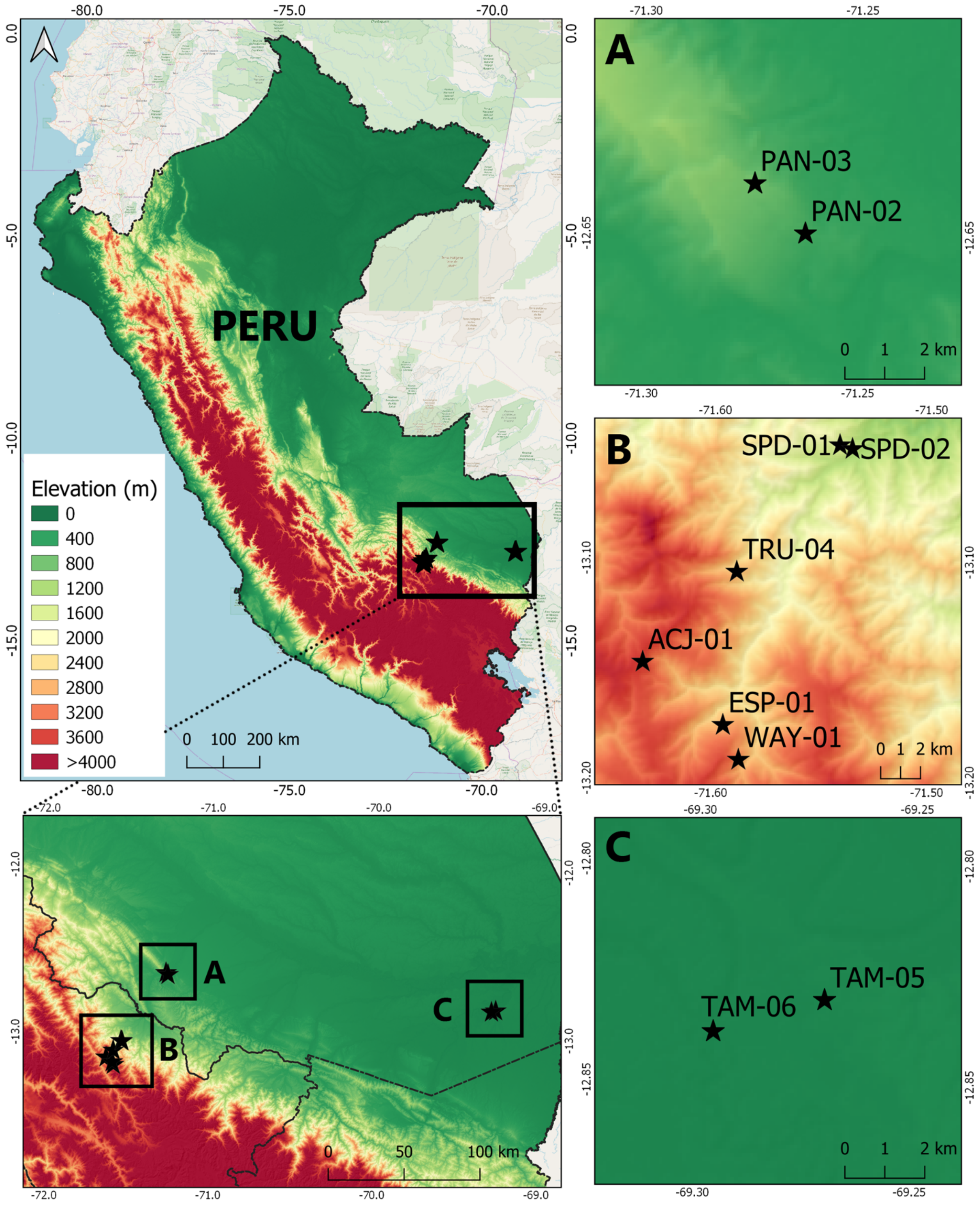

Table S1. Summary of number of individuals sampled and the taxonomic composition of the population are given.

Site	Individuals	Species	Genera	Families
Tambopata; TAM-06	36	15	15	11
Tambopata; TAM-05	55	26	21	15
Pantiacolla; PAN-02	25	13	11	10
Pantiacolla; PAN-03	26	13	11	8
San Pedro; SPD-02	53	23	23	19
San Pedro; SPD-01	46	25	22	19
Trocha Union; TRU-04	52	16	11	10
Esperanza; ESP-01	42	13	9	9
Wayquecha; WAY-01	19	10	7	6
Acjanaco; ACJ-01	31	9	7	7
**Combined total**	**385**	**134**	**89**	**49**

Table S2. Mean and standard error values for 18 canopy foliar traits on a mass-basis and LMA (leaf mass per unit area) in sun (bold) and shade leaves from 10 forest sites along an Andes-Amazon elevation gradient in Peru.

Trait	TAM-06	TAM-05	PAN-02	PAN-03	SPD-02	SPD-01	TRU-04	ESP-01	WAY-01	ACJ-01
					Light capture and growth					
**LMA (g m^{-2})**	**94.1±3.3***	**99.9±3.1***	**99.0±4.9***	**100.0±5.3**	**108.2±4.9***	**130.7±5.3***	**147.4±6.9***	**128.6±4.9***	**139.9±8.4**	**142.9±7.8***
	80.1±2.9	86.8±2.5	78.1±4.2	91.0±4.7	91.2±4.7	112.1±4.8	121.4±5.7	101.5±3.9	119.0±7.3	117.4±7.5
**N (%)**	**2.45±0.09**	**2.27±0.08**	**2.36±0.09**	**2.33±0.12**	**2.41±0.07**	**1.82±0.07**	**1.96±0.06**	**1.75±0.07**	**1.64±0.08**	**1.93±0.06**
	2.54±0.09	2.27±0.08	2.46±0.12	2.34±0.10	2.47±0.07	1.90±0.07	2.04±0.06	1.72±0.06	1.62±0.10	1.95±0.06
**Chl *ab* (mg g^{-1})**	**7.0±0.3***	**6.7±0.3***	**5.9±0.5***	**5.5±0.4***	**7.8±0.3***	**5.2±0.3***	**5.1±0.3***	**6.7±0.3***	**5.5±0.4***	**5.2±0.2***
	8.2±0.3	7.9±0.3	8.0±0.5	6.9±0.5	9.6±0.4	6.4±0.3	6.3±0.4	8.2±0.4	6.8±0.4	6.5±0.3
**Car (mg g^{-1})**	**1.5±0.1***	**1.5±0.1**	**1.2±0.1***	**1.2±0.1**	**1.7±0.1***	**1.2±0.1***	**1.1±0.1**	**1.5±0.1**	**1.3±0.1**	**1.2±0.0***
	1.6±0.0	1.7±0.1	1.5±0.1	1.4±0.1	1.9±0.1	1.4±0.1	1.3±0.1	1.7±0.1	1.5±0.1	1.3±0.1
**NSC (%)**	**48.0±1.8**	**42.5±1.1**	**42.5±1.8**	**45.1±2.6**	**49.6±1.2**	**46.1±1.6**	**56.2±1.5**	**52.2±1.7**	**49.9±1.8**	**61.7±1.7**
	46.1±1.8	40.9±1.1	41.2±1.7	42.8±2.6	48.3±1.3	44.5±1.6	56.6±1.6	51.8±1.4	50.2±1.8	61.6±1.5
**δ^{13}C (‰)**	**-30.9±0.3***	**-31.5±0.2***	**-30.8±0.2***	**-30.1±0.3***	**-30.0±0.2***	**-30.2±0.2***	**-28.6±0.3***	**-28.7±0.3***	**-28.6±0.5***	**-28.2±0.2***
	-32.0±0.3	-32.5±0.1	-32.6±0.3	-31.5±0.3	-31.3±0.2	-31.3±0.2	-29.9±0.2	-30.6±0.3	-30.2±0.4	-30.2±0.2
					Structure and defense					
**Total C (%)**	**47.9±0.6**	**50.3±0.4**	**50.2±0.5**	**51.2±0.4**	**49.9±0.3**	**50.4±0.4**	**51.9±0.3**	**47.8±0.4**	**50.2±0.7**	**47.8±0.3**
	47.3±0.6	49.5±0.5	49.6±0.6	51.2±0.5	49.5±0.3	50.1±0.3	51.5±0.3	47.2±0.5	49.2±0.5	47.6±0.3
**Lignin (%)**	**21.9±1.9**	**27.0±1.2**	**29.3±1.9**	**24.2±2.2**	**23.4±1.2**	**26.2±1.2**	**21.8±1.2**	**23.9±1.2**	**25.8±1.8**	**16.2±1.4**
	22.8±2.0	27.2±1.1	28.2±1.8	26.2±2.1	23.4±1.3	26.0±1.2	20.3±1.2	22.6±1.0	24.3±1.5	15.4±1.3
**Cellulose (%)**	**16.1±0.7**	**18.3±0.5**	**18.3±1.2**	**17.9±1.2**	**14.9±0.7**	**16.5±0.7**	**11.3±0.4**	**15.6±0.8**	**16.1±1.1**	**11.2±0.6**
	16.4±0.8	19.5±0.5	21.0±1.6	19.5±1.1	16.0±0.6	18.2±0.7	12.1±0.5	16.6±0.7	16.4±1.0	11.8±0.6
**Phenols (mg g^{-1})**	**48.1±6.9**	**86.4±6.1**	**55.0±7.3**	**70.1±11.9**	**88.0±6.9**	**89.6±6.1**	**123.0±5.3**	**109.1±5.9**	**134.5±7.1**	**64.7±7.2**
	50.7±7.6	82.1±6.9	57.4±8.2	63.6±11.0	85.4±6.4	82.3±6.9	119.7±6.0	101.1±7.2	127.4±6.8	55.6±6.3
**Tannins (mg g^{-1})**	**17.3±2.5**	**41.0±2.9**	**27.2±3.6**	**22.8±4.7**	**34.5±2.9**	**38.7±2.9**	**58.7±3.5**	**55.7±3.5**	**72.4±4.5**	**30.7±3.0**
	17.2±2.9	41.4±3.4	29.5±4.2	19.7±3.5	37.3±2.9	35.8±3.1	57.9±4.0	53.1±3.8	68.6±4.1	29.9±2.5

Asterisk indicates a statistical difference between a given foliar trait from sun or shade canopy position within the site ($p < 0.05$). LMA, N, chlorophyll *ab*, and carotenoids were log-transformed before analysis.

Table S2 (Cont.). Mean and standard error values for 18 canopy foliar traits on a mass-basis and LMA (leaf mass per unit area) in sun (bold) and shade leaves from 10 forest sites along an Andes-Amazon elevation gradient in Peru.

Trait	TAM-06	TAM-05	PAN-02	PAN-03	SPD-02	SPD-01	TRU-04	ESP-01	WAY-01	ACJ-01
					Macronutrients					
**P (%)**	**0.144±0.009**	**0.099±0.004**	**0.112±0.007**	**0.098±0.009**	**0.162±0.006**	**0.126±0.007**	**0.166±0.009**	**0.114±0.005**	**0.155±0.010**	**0.139±0.006**
	0.155±0.010	0.100±0.004	0.123±0.010	0.092±0.007	0.168±0.006	0.130±0.006	0.170±0.008	0.117±0.006	0.155±0.010	0.149±0.008
**Ca (%)**	**1.31±0.09**	**0.28±0.03**	**0.15±0.01**	**0.09±0.01**	**0.91±0.06**	**0.56±0.05**	**0.38±0.03**	**0.78±0.06**	**0.58±0.04**	**0.51±0.04**
	1.38±0.09	0.31±0.03	0.16±0.01	0.10±0.01	0.89±0.06	0.51±0.04	0.41±0.03	0.89±0.07	0.63±0.04	0.52±0.04
**K (%)**	**0.72±0.06**	**0.69±0.05**	**0.57±0.03**	**0.49±0.04**	**0.55±0.02***	**0.47±0.03**	**0.66±0.03***	**0.66±0.03**	**0.66±0.04**	**0.70±0.05**
	0.79±0.06	0.76±0.05	0.66±0.04	0.49±0.03	0.66±0.04	0.54±0.04	0.75±0.03	0.75±0.05	0.71±0.05	0.83±0.07
**Mg (%)**	**0.35±0.03**	**0.24±0.02**	**0.19±0.01***	**0.14±0.01**	**0.37±0.02**	**0.26±0.02**	**0.27±0.02**	**0.32±0.02**	**0.33±0.03**	**0.27±0.02**
	0.38±0.03	0.27±0.02	0.22±0.01	0.15±0.01	0.37±0.02	0.26±0.02	0.29±0.02	0.35±0.02	0.35±0.03	0.28±0.02
					Micronutrients					
**B (µg g^{-1})**	**26.97±3.04**	**25.83±1.91**	**16.64±1.87**	**14.90±1.40**	**19.05±1.30**	**13.14±0.76**	**19.99±1.17**	**24.84±1.44**	**29.65±2.07**	**16.51±1.02**
	27.26±2.87	27.56±2.03	17.73±2.28	14.86±1.35	18.33±1.31	14.03±0.85	20.48±1.23	25.81±1.00	30.10±2.21	17.53±0.72
**Fe (µg g^{-1})**	**51.96±2.78**	**51.93±1.71**	**74.28±5.58**	**59.57±5.24**	**73.05±7.21**	**56.35±4.27**	**44.30±1.72**	**71.75±4.86***	**49.93±4.61**	**64.27±5.80**
	51.58±1.86	50.85±1.54	77.33±4.51	72.67±10.57	70.68±3.91	60.53±4.40	45.03±2.04	95.38±7.26	53.79±5.99	76.69±11.39
**Mn (µg g^{-1})**	**223.75±46.34**	**380.97±67.88**	**39.02±5.02**	**45.20±5.51**	**423.89±54.73**	**242.65±32.88**	**465.19±43.32**	**859.24±95.20**	**1418.59±143.54**	**755.41±46.62**
	195.79±37.21	398.41±66.48	44.79±5.81	45.61±4.35	356.99±44.87	213.62±26.32	472.65±47.03	852.43±85.03	1475.12±151.11	764.30±58.67
**Zn (µg g^{-1})**	**21.67±3.44**	**13.94±1.15**	**14.33±0.74**	**13.62±1.16**	**15.32±1.01**	**12.08±0.77**	**15.19±0.89**	**16.42±1.25**	**16.65±1.58**	**15.84±1.57**
	22.34±3.90	15.68±1.44	17.18±1.44	13.31±1.04	16.49±1.25	12.49±0.73	16.28±1.03	19.36±2.31	17.22±1.77	16.61±1.49

Asterisk indicates a statistical difference between a given foliar trait from sun or shade canopy position within the site ($p < 0.05$).
P, K, and Mg were log-transformed before analysis. Ca was transformed by square root.

Table S3. Mean and standard error values for 18 canopy foliar traits on an area-basis and SLA (specific leaf area; 1/LMA) in sun (bold) and shade leaves from 10 forest sites along an Andes-Amazon elevation gradient in Peru.

Trait	TAM-06	TAM-05	PAN-02	PAN-03	SPD-02	SPD-01	TRU-04	ESP-01	WAY-01	ACJ-01
					Light capture and growth					
**SLA (cm^2 g^{-1})**	**111.3±4.3***	**105.6±3.6***	**107.9±6.3***	**107.1±5.7**	**102.6±4.8***	**82.5±3.5***	**76.4±3.9***	**82.7±3.2***	**76.6±4.8**	**75.6±3.6***
	131.6±5.6	121.0±3.9	137.9±8.9	117.2±5.8	126.7±7.1	95.7±3.6	92.8±4.7	105.9±4.9	90.7±6.6	95.3±5.6
**N (g m^{-2})**	**2.29±0.10***	**2.22±0.08***	**2.18±0.14***	**2.24±0.17**	**2.55±0.11***	**2.29±0.08***	**2.80±0.13***	**2.17±0.07***	**2.23±0.12**	**2.68±0.11***
	2.01±0.09	1.94±0.08	1.78±0.12	2.10±0.12	2.17±0.10	2.07±0.08	2.39±0.10	1.68±0.06	1.82±0.18	2.19±0.10
**Chl *ab* (μg cm^{-2})**	**63.9±2.0**	**63.4±2.2**	**53.4±4.2**	**51.9±4.4**	**78.8±2.2**	**64.8±2.8**	**67.2±2.4**	**81.8±3.1**	**72.9±4.3**	**72.6±3.3**
	64.4±2.6	66.3±1.9	57.5±4.2	60.1±3.4	80.0±2.7	67.9±2.3	69.0±2.5	78.6±2.4	73.2±6.2	71.3±3.3
**Car (μg cm^{-2})**	**13.6±0.5**	**14.3±0.6**	**10.5±0.8**	**11.3±0.8**	**17.0±0.5**	**14.6±0.6**	**14.9±0.4**	**18.1±0.7***	**16.8±0.8**	**16.3±0.8**
	13.0±0.6	13.9±0.5	10.8±0.8	12.2±0.7	16.4±0.6	14.4±0.6	14.1±0.5	16.2±0.6	15.7±1.3	15.2±0.9
**NSC (g m^{-2})**	**45.7±2.7***	**42.2±1.6***	**40.1±3.1***	**41.9±2.9**	**53.6±2.9***	**60.5±3.4***	**81.1±3.8***	**67.1±3.3***	**69.9±5.1**	**86.3±3.9***
	37.1±2.1	35.3±1.3	31.1±2.6	37.8±2.1	43.6±2.5	49.7±2.6	67.7±3.3	52.7±2.7	56.9±5.6	71.1±4.0
**$\delta^{13}C$ (g m^{-2})**	**-2.90±0.10***	**-3.13±0.09***	**-3.04±0.14**	**-3.02±0.17**	**-3.23±0.14**	**-3.92±0.15***	**-4.16±0.18***	**-3.68±0.14***	**-3.93±0.19**	**-4.01±0.22**
	-2.56±0.09	-2.82±0.08	-2.53±0.13	-2.86±0.15	-2.85±0.15	-3.49±0.14	-3.63±0.16	-3.10±0.12	-3.56±0.18	-3.52±0.21
					Structure and defense					
**Total C (g m^{-2})**	**44.9±1.5***	**50.4±1.7***	**47.4±3.1***	**49.6±3.4**	**54.1±2.5***	**65.8±2.7***	**76.9±3.8***	**61.5±2.5***	**70.4±4.4***	**68.6±4.1***
	38.0±1.4	43.1±1.4	36.9±2.5	46.8±2.6	45.3±2.4	56.3±2.5	62.8±3.1	48.0±2.0	55.6±4.8	56.2±3.8
**Lignin (g m^{-2})**	**20.5±1.8**	**27.6±1.8**	**27.8±2.4***	**25.1±3.4**	**26.1±1.9**	**34.4±2.2**	**33.2±2.8***	**31.0±2.1***	**36.5±3.2***	**23.6±2.6**
	18.4±1.7	24.2±1.5	20.8±1.9	25.2±3.1	22.4±1.8	29.7±2.1	25.4±2.2	22.7±1.3	27.3±2.6	18.6±2.3
**Cellulose (g m^{-2})**	**14.9±0.7***	**18.2±0.8**	**17.8±1.8**	**17.4±1.6**	**16.1±1.0**	**21.3±1.3**	**17.1±1.2**	**20.1±1.3***	**22.3±1.7**	**16.3±1.5**
	12.9±0.7	16.9±0.7	15.8±2.0	17.8±1.4	14.7±1.0	20.1±1.2	15.1±1.1	16.9±1.0	18.3±1.8	14.0±1.3
**Phenols (g m^{-2})**	**46.4±6.5**	**90.1±6.7***	**54.4±9.1**	**62.9±9.3**	**98.6±8.5**	**119.4±9.8**	**171.6±6.9***	**141.3±9.2***	**182.2±10.4***	**96.8±12.6**
	42.3±6.2	74.4±6.5	45.2±8.2	55.0±8.8	81.9±7.5	96.1±9.7	140.6±8.3	106.0±8.5	139.7±13.1	73.8±10.9
**Tannins (g m^{-2})**	**16.6±2.3**	**42.3±3.1**	**26.9±4.5**	**21.0±3.6**	**38.2±3.5**	**50.9±4.2**	**81.2±4.5***	**72.1±5.3***	**98.1±7.1***	**45.2±5.3**
	14.5±2.4	36.9±3.0	23.0±3.9	17.1±2.7	34.2±2.9	40.6±4.1	67.0±4.7	54.9±4.6	75.3±7.8	36.9±3.8

Asterisk indicates a statistical difference between a given foliar trait from sun or shade canopy position within the site ($p < 0.05$).
LMA, N, chlorophyll *ab*, and carotenoids were log-transformed before analysis.

Table S3 (Cont.). Mean and standard error values for 18 canopy foliar traits on an area-basis and SLA (specific leaf area; 1/LMA) in sun (bold) and shade leaves from 10 forest sites along an Andes-Amazon elevation gradient in Peru.

Trait	TAM-06	TAM-05	PAN-02	PAN-03	SPD-02	SPD-01	TRU-04	ESP-01	WAY-01	ACJ-01
					Macronutrients					
**P (g m^{-2})**	**0.136±0.010**	**0.095±0.004***	**0.098±0.005***	**0.090±0.007**	**0.170±0.008***	**0.161±0.010**	**0.230±0.012***	**0.141±0.006***	**0.216±0.020**	**0.191±0.009***
	0.123±0.008	0.085±0.004	0.084±0.006	0.080±0.005	0.147±0.008	0.145±0.009	0.194±0.010	0.113±0.004	0.174±0.016	0.164±0.009
**Ca (g m^{-2})**	**1.26±0.11**	**0.27±0.03**	**0.15±0.01**	**0.09±0.01**	**1.03±0.12**	**0.74±0.07**	**0.53±0.03**	**0.98±0.08**	**0.82±0.08**	**0.69±0.05**
	1.11±0.09	0.26±0.03	0.12±0.01	0.08±0.01	0.86±0.10	0.59±0.06	0.46±0.03	0.86±0.06	0.73±0.08	0.59±0.04
**K (g m^{-2})**	**0.66±0.05**	**0.65±0.04**	**0.53±0.04**	**0.46±0.04**	**0.58±0.03**	**0.59±0.04**	**0.91±0.04**	**0.81±0.03***	**0.90±0.06**	**0.97±0.06**
	0.62±0.05	0.62±0.03	0.47±0.04	0.43±0.03	0.56±0.04	0.59±0.04	0.85±0.03	0.71±0.03	0.79±0.07	0.91±0.07
**Mg (g m^{-2})**	**0.32±0.02**	**0.22±0.01**	**0.18±0.01**	**0.12±0.01**	**0.38±0.02**	**0.34±0.03**	**0.38±0.02**	**0.39±0.03**	**0.45±0.04**	**0.37±0.03**
	0.30±0.02	0.22±0.01	0.16±0.01	0.13±0.01	0.32±0.02	0.30±0.02	0.33±0.02	0.33±0.02	0.39±0.05	0.30±0.02
					Micronutrients					
**B (ng cm^{-2})**	**46.47±0.03**	**46.68±0.02**	**38.01±0.02**	**36.85±0.02**	**58.01±0.01**	**47.28±0.01**	**49.98±0.01**	**59.88±0.02**	**54.08±0.03**	**51.96±0.01**
	46.56±0.02	48.18±0.02	40.60±0.02	42.29±0.01	58.09±0.01	49.16±0.01	50.88±0.01	56.92±0.01	53.53±0.03	50.53±0.01
**Fe (ng cm^{-2})**	**17.39±0.03**	**16.73±0.02**	**15.35±0.07**	**15.05±0.05**	**20.76±0.06**	**17.51±0.06**	**17.19±0.03**	**21.93±0.07**	**18.84±0.07**	**20.66±0.10**
	17.88±0.02	18.12±0.02	16.86±0.04	17.84±0.07	21.92±0.05	18.76±0.06	18.11±0.02	21.63±0.07	19.64±0.08	20.78±0.12
**Mn (ng cm^{-2})**	**0.25±0.33**	**0.25±0.66**	**0.16±0.05**	**0.14±0.05**	**0.20±0.69**	**0.17±0.42**	**0.27±0.63**	**0.32±1.60**	**0.40±2.76**	**0.23±0.94**
	0.21±0.26	0.23±0.53	0.13±0.05	0.13±0.03	0.15±0.45	0.15±0.30	0.22±0.52	0.26±1.05	0.33±2.46	0.20±1.04
**Zn (ng cm^{-2})**	**0.49±0.03**	**0.51±0.01**	**0.69±0.01**	**0.56±0.01**	**0.75±0.01**	**0.72±0.01**	**0.62±0.01**	**0.92±0.01**	**0.68±0.04**	**0.93±0.03**
	0.41±0.03	0.43±0.01	0.55±0.01	0.62±0.01	0.63±0.01	0.68±0.01	0.51±0.01	0.94±0.02	0.61±0.03	0.87±0.02

Asterisk indicates a statistical difference between a given foliar trait from sun or shade canopy position within the site ($p < 0.05$).
P, K, and Mg were log-transformed before analysis. Ca was transformed by square root.

Table S4. Results of nested ANOVA testing for differences among leaf traits on an area-basis between sun and shade leaves and site[1]. Mean offset among canopy position across all sites is also shown.

	Source of variation				
	Site		Canopy position (site)		Offset
**Response variable**	**F**	***P***	**F**	***P***	**μ±STERR**
	Light capture and growth				
**N**	8.60	< 0.01	6.29	< 0.01	-0.34+0.02*
**Chlorophyll *ab***	15.43	< 0.01	0.57	NS	
**Carotenoids**	17.55	< 0.01	0.99	NS	
**NSC**	47.30	< 0.01	6.87	< 0.01	-10.52+0.68*
**$\delta^{13}C$**	17.47	< 0.01	4.16	< 0.01	0.42+0.03*
	Structure and defense				
**C**	23.06	< 0.01	7.70	< 0.01	-9.94+0.58*
**Lignin**	8.02	< 0.01	2.65	< 0.01	-4.98+0.39*
**Cellulose**	8.31	< 0.01	1.74	NS	
**Phenols**	18.04	< 0.01	1.61	NS	
**Tannins**	31.57	< 0.01	1.59	NS	
	Macronutrients				
**P**	72.18	< 0.01	4.28	< 0.01	-0.021+0.002*
**Ca**	91.91	< 0.01	1.46	NS	
**K**	31.33	< 0.01	0.84	NS	
**Mg**	38.21	< 0.01	1.51	NS	
	Micronutrients				
**B**	23.99	< 0.01	0.24	NS	
**Fe**	23.39	< 0.01	1.63	NS	
**Mn**	68.02	< 0.01	0.08	NS	
**Zn**	5.01	< 0.01	0.52	NS	

[1]Sample collection at sites varying in elevation, light environment, and geology meant that canopy position was effectively nested within site. Non-statistically significant results are indicated by NS. N, soluble-C, water, P, K, Mg, C, lignin, cellulose, phenols and tannins were log-transformed before analysis. Ca was transformed by square root. STERR is standard error. * indicates significant offset at $p < 0.01$.

Table S5. Relationships between site-level mean leaf traits on an area basis from the sun or shade layer of the canopy and elevation. Correlation value (R^2) is provided with root mean squared error (RMSE) in parentheses. The equations are reported relative to elevation in km.

	Sun layer		Shade layer	
**Trait**	**R^2**	**Equation**	**R^2**	**Equation**
		Light capture and growth		
LMA	0.87 (7.9)***	15.6 x Elevation + 92.1	0.78 (8.3)***	11.8 x Elevation + 79.6
N	NS		NS	
Chlorophyll *ab*	0.42 (7.9)*	5.1 x Elevation + 58.3	NS	3.6 x Elevation + 62.7
Carotenoids	0.49 (1.9)*	1.4 x Elevation + 12.3	0.43 (1.4)*	0.9 x Elevation + 12.6
NSC	0.89 (6.0)***	12.7 x Elevation + 36.9	0.86 (5.5)***	10.3 x Elevation + 30.6
$\delta^{13}C$	0.79 (0.2)***	-0.3 x Elevation + -2.9	0.71 (0.2)**	-0.3 x Elevation + -2.6
		Structure and defense		
C	0.77 (5.6)***	7.9 x Elevation + 45.4	0.63 (5.5)**	5.5 x Elevation + 39.5
Lignin	NS		NS	
Cellulose	NS		NS	
Phenols[1]	0.95 (13.0)***	47.0 x Elevation + 30.4	0.91 (12.6)***	34.4 x Elevation + 30.4
Tannins[1]	0.94 (8.2)***	26.7 x Elevation + 5.5	0.93 (6.5)***	20.2 x Elevation + 6.7
		Macronutrients		
P	0.62 (0.03)**	0.03 x Elevation + 0.10	0.54 (0.03)*	0.0 x Elevation + 0.1
Ca	NS		NS	
K[1]	0.69 (0.11)**	0.07*elev2-0.14*elev+0.63	0.65 (0.10)**	0.07*elev2-0.18*elev+0.27
Mg	0.53 (0.08)*	0.06 x Elevation + 0.21	0.46 (0.07)*	0.0 x Elevation + 0.2
		Micronutrients		
B[1]	0.54 (0.07)*	0.06*elev2-0.12*elev+0.59	0.52 (0.05)*	0.06*elev2-0.15*elev+0.23
Fe	0.57 (0.10)*	0.09 x Elevation + 0.53	0.51 (0.13)*	0.1 x Elevation + 0.5
Mn	0.63 (4.1)**	4.1 x Elevation + -0.6	0.61 (3.6)**	3.4 x Elevation + -0.6
Zn	0.60 (0.03)**	0.03 x Elevation + 0.13	0.52 (0.03)*	0.0 x Elevation + 0.1

Asterisks indicate significant levels as * $p < 0.001$, ** $p < 0.01$, and *** $p < 0.05$. [1]sites ACJ-01 and TAM-05 excluded from regressions due to unusual soil properties interacting with these traits. See methods for details.

Table S6. Sources of variation (coefficient of variation) in mass-based foliar traits within each site are listed in order of sun versus shade within species within site, intra-specific and inter-specific variation for 19 canopy foliar traits from 10 forest sites along an Andes-Amazon elevation gradient in Peru.

Trait	TAM-06	TAM-05	PAN-02	PAN-03	SPD-02	SPD-01	TRU-04	ESP-01	WAY-01	ACJ-01
					Light capture and growth					
**LMA (g m^{-2})**	12.2|15.3|16.8	10.5|13.3|22.8	17.2|17.7|24.2	7.3|11.5|24.1	14.9|17.8|35.6	11.5|14.0|26.7	14.4|16.1|34.3	18.3|21.4|20.7	19.3|19.8|23.3	15.9|16.3|30.7
**N (%)**	5.7|8.7|19.8	4.0|6.8|23.4	4.7|6.2|22.2	6.0|7.4|21.5	5.1|6.7|20.6	6.1|6.5|26.3	6.3|8.2|20.9	5.4|8.4|20.9	8.1|9.1|25.5	4.9|7.2|15.1
**Chl *ab* (mg g^{-1})**	13.5|15.1|17.8	18.6|22.3|27.3	25.7|24.8|29.5	23.4|24.8|24.8	18.2|23.4|27.0	20.8|22.0|28.1	21.4|25.1|35.9	19.7|22.6|24.5	25.2|25.8|29.7	18.9|19.5|15.3
**Car (mg g^{-1})**	11.4|12.7|17.4	13.9|17.0|30.7	23.4|21.3|27.9	16.7|18.6|24.6	15.4|19.4|23.8	17.3|17.8|30.1	16.7|19.8|36.6	17.0|19.7|27.6	18.6|18.5|29.1	15.6|16.7|16.3
**NSC (%)**	4.6|6.5|21.5	4.7|6.2|19.8	5.0|5.5|21.7	4.5|7.8|33.7	3.4|5.1|18.4	5.3|7.0|21.5	4.4|6.1|21.0	6.3|8.2|15.6	4.0|4.8|13.1	5.3|7.4|13.0
**δ^{13}C (‰)**	-2.7|-3.4|-4.7	-2.4|-2.7|-3.2	-4.0|-4.0|-3.8	-3.2|-3.9|-4.7	-3.4|-3.9|-3.6	-3.1|-4.1|-3.8	-4.0|-4.6|-4.4	-4.6|-4.4|-5.4	-5.8|-5.1|-5.1	-4.9|-4.3|-2.1
					Structure and defense					
**Total C (%)**	1.4|2.2|6.8	1.5|2.0|6.8	0.9|1.4|5.4	0.8|1.6|4.1	1.0|1.5|5.2	1.1|1.1|4.4	0.9|1.6|4.0	1.1|1.5|5.6	2.1|2.4|6.5	0.9|1.2|3.1
**Lignin (%)**	6.8|10.0|46.8	5.4|7.9|33.6	17.0|21.2|25.6	8.0|11.0|39.4	8.7|11.8|39.5	5.9|8.7|27.7	8.7|11.2|47.4	12.3|13.7|23.2	10.9|11.6|28.2	10.5|18.5|43.3
**Cellulose (%)**	11.2|16.6|23.8	6.5|7.8|18.7	19.7|28.4|22.7	8.5|10.1|29.3	9.1|10.4|27.4	10.4|12.7|25.2	6.8|9.2|30.0	9.4|9.8|26.8	8.0|9.3|26.8	8.7|14.2|20.6
**Phenols (%)**	29.5|49.6|74.4	37.3|38.7|50.6	46.9|52.4|75.0	41.4|65.6|93.4	27.0|30.4|49.4	21.8|22.4|50.8	12.1|20.2|32.1	21.6|23.1|32.5	13.1|15.8|19.9	31.8|50.2|51.2
**Tannins (%)**	39.6|65.7|82.0	35.0|38.9|51.6	45.5|53.3|76.0	59.4|86.1|96.7	25.6|30.3|47.3	22.9|25.2|48.4	14.6|25.2|42.9	19.2|22.1|35.3	10.8|16.2|26.5	26.3|40.6|35.4
					Macronutrients					
**P (%)**	9.4|13.4|31.6	6.6|9.8|31.1	11.2|15.0|31.1	6.2|15.0|28.9	8.5|10.4|33.8	8.1|10.1|33.9	10.5|15.1|33.8	7.9|12.2|25.0	10.8|14.9|23.8	9.1|13.4|21.4
**Ca (%)**	13.4|21.5|34.6	12.8|21.8|76.1	15.3|18.9|30.8	15.4|29.3|35.1	12.6|20.2|57.2	17.0|19.3|53.0	12.6|20.7|65.8	17.3|27.7|40.6	12.6|16.5|27.1	12.2|16.0|36.8
**K (%)**	12.6|20.7|40.4	11.9|16.5|46.7	14.3|16.8|33.5	9.7|18.6|30.3	15.9|20.5|34.1	12.4|18.6|43.1	13.4|16.3|26.4	16.8|20.6|31.1	17.2|23.7|21.3	11.2|18.4|30.9
**Mg (%)**	9.8|13.5|47.1	12.1|20.0|50.4	13.9|14.8|21.7	12.9|18.6|32.5	10.5|15.6|42.5	10.2|12.2|42.7	9.6|13.5|67.7	11.1|18.2|44.8	9.0|15.3|37.6	9.3|15.4|46.4
					Micronutrients					
**B (µg g^{-1})**	9.9|23.8|60.7	12.1|17.4|54.3	15.1|19.7|54.0	17.2|21.5|39.0	14.2|17.9|48.4	12.2|16.5|39.8	13.0|20.4|36.6	15.6|21.2|25.4	11.3|16.8|29.8	11.4|18.3|27.0
**Fe (µg g^{-1})**	12.0|15.4|23.0	10.0|13.0|20.5	13.7|18.6|26.9	16.0|21.3|72.5	15.9|19.6|64.0	17.2|24.2|35.2	9.7|13.7|24.9	20.8|26.1|32.3	16.6|22.9|36.5	15.6|29.0|54.3
**Mn (µg g^{-1})**	16.6|30.0|111.2	20.4|30.3|121.5	17.3|26.9|43.8	18.7|29.8|47.3	15.4|28.2|79.1	18.4|19.6|80.3	15.5|24.2|74.6	21.4|30.5|62.5	14.6|17.4|41.8	14.4|20.4|46.9
**Zn (µg g^{-1})**	12.8|19.3|89.8	11.1|15.5|55.5	14.4|14.9|29.8	9.2|15.2|33.8	10.1|14.8|59.2	11.2|14.3|39.0	10.3|14.0|57.8	12.1|17.5|49.8	10.4|13.7|43.3	8.4|13.3|48.3
